# Machine learning gene expression predicting model for ustekinumab response in patients with Crohn's disease

**DOI:** 10.1002/iid3.506

**Published:** 2021-09-01

**Authors:** Manrong He, Chao Li, Wanxin Tang, Yingxi Kang, Yongdi Zuo, Yufang Wang

**Affiliations:** ^1^ Department of Nephrology, West China Hospital Sichuan University Chengdu Sichuan China; ^2^ Department of Gastroenterology, West China Hospital Sichuan University Chengdu Sichuan China

**Keywords:** Crohn's disease, LASSO regression, machine learning model, ustekinumab

## Abstract

**Background:**

Recent studies reported the responses of ustekinumab (UST) for the treatment of Crohn's disease (CD) differ among patients, while the cause was unrevealed. The study aimed to develop a prediction model based on the gene transcription profiling of patients with CD in response to UST.

**Methods:**

The GSE112366 dataset, which contains 86 CD and 26 normal samples, was downloaded for analysis. Differentially expressed genes (DEGs) were identified first. Gene Ontology (GO) and Kyoto Encyclopedia of Genes and Genomes (KEGG) pathway analyses were administered. Least absolute shrinkage and selection operator regression analysis was performed to build a model for UST response prediction.

**Results:**

A total of 122 DEGs were identified. GO and KEGG analyses revealed that immune response pathways are significantly enriched in patients with CD. A multivariate logistic regression equation that comprises four genes (*HSD3B1, MUC4, CF1*, and *CCL11*) for UST response prediction was built. The area under the receiver operator characteristic curve for patients in training set and testing set were 0.746 and 0.734, respectively.

**Conclusions:**

This study is the first to build a gene expression prediction model for UST response in patients with CD and provides valuable data sources for further studies.

## Graphical abstract

In the present study, we used bioinformatics analysis to obtain the gene expression profiles from the Gene Expression Omnibus to build a model for UST response prediction. As a result, the gene expression profiling was revealed, and a multivariate logistic regression equation that comprises four genes (HSD3B1, MUC4, CF1, and CCL11) for UST response prediction was successfully built and evaluated. This study is the first to build a machine learning gene expression prediction model for UST response in patients with CD and provides valuable data sources for further basic and clinical studies in the future.

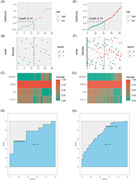

## 1 INTRODUCTION

Inflammatory bowel diseases (IBDs), composed of Crohn's disease (CD) and ulcerative colitis (UC), are chronic, progressive, and recurring diseases that threaten human health.^1^ Any part of the gastrointestinal tract and all layers of the mucosal wall could be damaged by CD. Intestinal stenosis or penetration occurs in CD progression in at least 50% of patients. CD is considered a heterogeneous disease with multiple etiologies, of which the main feature is immune response to various microbial antigens.^2^, ^3^, ^4^, ^5^ The pathogenesis of CD has not yet been fully clarified. Some specific genes concerning CD have been reported recently. For example, NOD2 is related to bacterial sensing; ATG16L1 is associated with inflamed terminal ileum; and MUC1, MUC2, and MUC4 are connected to the dysregulation of the key epithelial barrier and innate immunity.^6^, ^7^, ^8^

The main strategies in CD treatment are the introduction of corticosteroids, immunosuppression (thiopurines and methotrexate), or combination therapy with biologicals (antitumor necrosis factor [TNF] and antiadhesion molecules) in high‐risk patients in addition to frequent inflammation control.^9^, ^10^, ^11^, ^12^ Anti‐TNF therapy symbolizes an important milestone particularly advanced in the clinical management of moderate to severe CD.^13^, ^14^ However, patients with primary nonresponse, secondary loss of response, or unbearable side effects to conventional treatment and TNF antagonists require other alternative treatment regimens.^15^ The monoclonal antibody ustekinumab (UST) is an inhibitor of the p40 subunit shared by proinflammatory cytokines, interleukin (IL)−12 and IL‐23, that further dampens the inflammatory cascade and the differentiation of inflammatory T cells. Clinical trials and clinical practice have demonstrated the efficacy and safety of UST for anti‐TNF‐naive and anti‐TNF‐exposed patients.^16^, ^17^, ^18^, ^19^, ^20^ Emerging data suggested that microbiome composition may be a marker of UST response. Validated serological and genetic markers of response to these agents are currently lacking.^21^ Nevertheless, some patients are unresponsive to UST.^21^ Unresponsiveness to UST could be attributed to high placebo rate and insufficient UST induction dose.^17^

Sporadic reports are far from revealing the treatment effect of UST in patients with CD. Additionally, few studies have assessed the responsiveness of patients to UST. We envisage that drug responsiveness may be related to genes. Accordingly, the purpose of this study was to analyze the expression of genes related to UST response by bioinformatic analysis. Bioinformatic analysis is a critical and scientific method for processing large amounts of data and acquiring valuable information. Bioinformatics has been widely used in many fields, such as the study of lupus nephritis, renal cell carcinoma, and oral squamous cell carcinoma.^22^, ^23^, ^24^, ^25^, ^26^ Few studies have used bioinformatic analysis to characterize UST response in patients with CD. The present study used the Gene Expression Omnibus (GEO) database to perform full gene transcription profiling in patients with CD, develop a machine learning model for predicting UST response, and provide valuable data resources for future research.

## 2 METHOD

### 2.1 Data retrieval

The transcription dataset was searched from the GEO database. The GSE112366 dataset, which contains 388 samples, including 362 patient samples with CD and 26 normal control samples, was retrieved. The effectiveness of UST induction was evaluated in patients with CD who have failed conventional treatments. In our study, we selected cases who were treated with UST 90 mg q8w. Terminal ileum tissues were taken before treatment for transcriptome sequencing. After treatment for 8 weeks, the patients were evaluated for a UST response. UST‐induced responders were defined as a reduction in Crohn's disease activity index ≥100.^27^ Eighty‐six samples from the CD group met the criteria. Then, we downloaded the corresponding expression matrix and matched clinical information.

### 2.2 Analysis of differentially expressed genes (DEGs)

DEGs were analyzed by the Limma package (version 3.42.0) of R 25 after data preprocessing. The adjusted *p* value and fold change (FC) were calculated by the linear fit method, Bayesian analysis, and *t* test algorithm. The cut‐off values for significant DEGs were |log2(FC)|>1 and adjusted *p* < .05. The ggplot2 (version 3.3.1) software package was used for visualization.

### 2.3 Gene set enrichment analysis (GSEA)‐based Kyoto Encyclopedia of Genes and Genomes (KEGG) pathway analysis

GSEA can identify functional enrichment by comparison of genes with predefined gene sets. A gene set is a group of genes, which shares localization, pathways, functions, or other features. The clusterProfiler package (version 3.5) was used to conduct GSEA. The FC of gene expression was subsequently calculated between the CD group and the control group, and based on the change of |log2(FC)|, the gene list was generated. Then, GSEA‐based KEGG analysis was conducted using the gseKEGG function in the clusterProfiler package. Adjusted *p* < .05 was set as the cut‐off criteria.

### 2.4 Gene Ontology (GO) enrichment analysis of significant DEGs

The GO analysis encompassed three independent domains: biological process (BP), cellular component (CC), and molecular function (MF). In this study, GO enrichment analysis of the identified significant DEGs was performed using the clusterProfiler package (version 3.5). Only GO term with adjusted *p* < .05 was considered significantly enriched.

### 2.5 Univariate logistic analysis

Univariate logistic regression analysis between significant DEGs and UST response was performed using the fitting generalized linear model function of R studio with the major augment “family = binomial” to determine UST response‐associated genes. Then, hazard ratio (HR), 95% confidence interval (95% CI), and *p* value were calculated. The results of the univariate logistic analysis were visualized as random forest plot by using “forestplot” R package (version 1.9).

### 2.6 Samples splitting

The “Handout” method was used for splitting samples. In detail, all samples were randomly split into a training set and a testing set by using the classification and regression training (caret) package (version 6.0‐85). Briefly, the samples were divided into the training and testing sets at a ratio of 70%:30% using the “createDataPartition” function in the R package “caret” to keep the data distribution of the training and testing sets consistent.

### 2.7 Construction of multivariate predictive model using least absolute shrinkage and selection operator (LASSO) regression

We applied LASSO regression to gain the final important predictors related to UST response. This process, which is one of machine learning methods adopted in several studies, was performed using the glmnet package (version 3.0‐2) in R. A multivariate regression formula was built based on the gene expression value of significant DEGs and UST response events under the training set. Finally, several predictors of significant DEGs with nonzero LASSO coefficients were obtained. Thus, a multivariate predictive model was constructed.

### 2.8 Evaluation of the multivariate predictive model

We built receiver operator characteristic (ROC) curves using the pROC R package (version 1.16.1) to assess the efficiency of the multivariate predictive model. Similarly, we performed the same processes in the testing group and the total dataset to evaluate the efficiency of the multivariate predictive model constructed by LASSO regression.

### 2.9 Statistics analysis

DEG, univariate logistic regression, LASSO regression, ROC, GSEA‐based KEGG, and GO analyses were performed using the R‐studio platform (v. 3.5.1). Adjusted *p* < .05 was considered statistically significant difference. All involved R software packages have been described previously.

## 3 RESULTS

### 3.1 Workflow of the study

**Figure 1:**
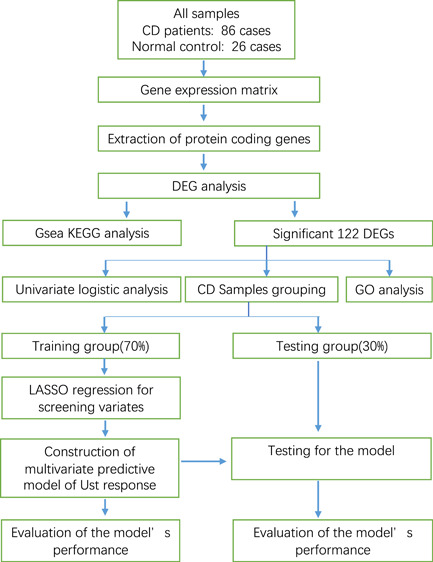
Workflow of the study

Figure 1 shows our workflow. A total of 112 legal samples from the GSE112366 dataset, including 86 CD cases and 26 normal control, were used in this study. The expression data of protein‐coding genes were extracted from the gene expression matrix, and then differential gene analysis was performed. Based on GSEA, GO and KEGG analyses were conducted on the DEGs. The most significant 122 DEGs (|FC|>2 and adjusted *p* < .05) were screened out for univariate logistic analysis and regression analysis. The CD samples were divided into a training set and a testing set at a ratio of 70%:30%. We built a multivariate predictive model of UST response in the training set first and then evaluated the model's performance in the testing set.

### 3.2 GSEA‐based KEGG analysis

**Figure 2:**
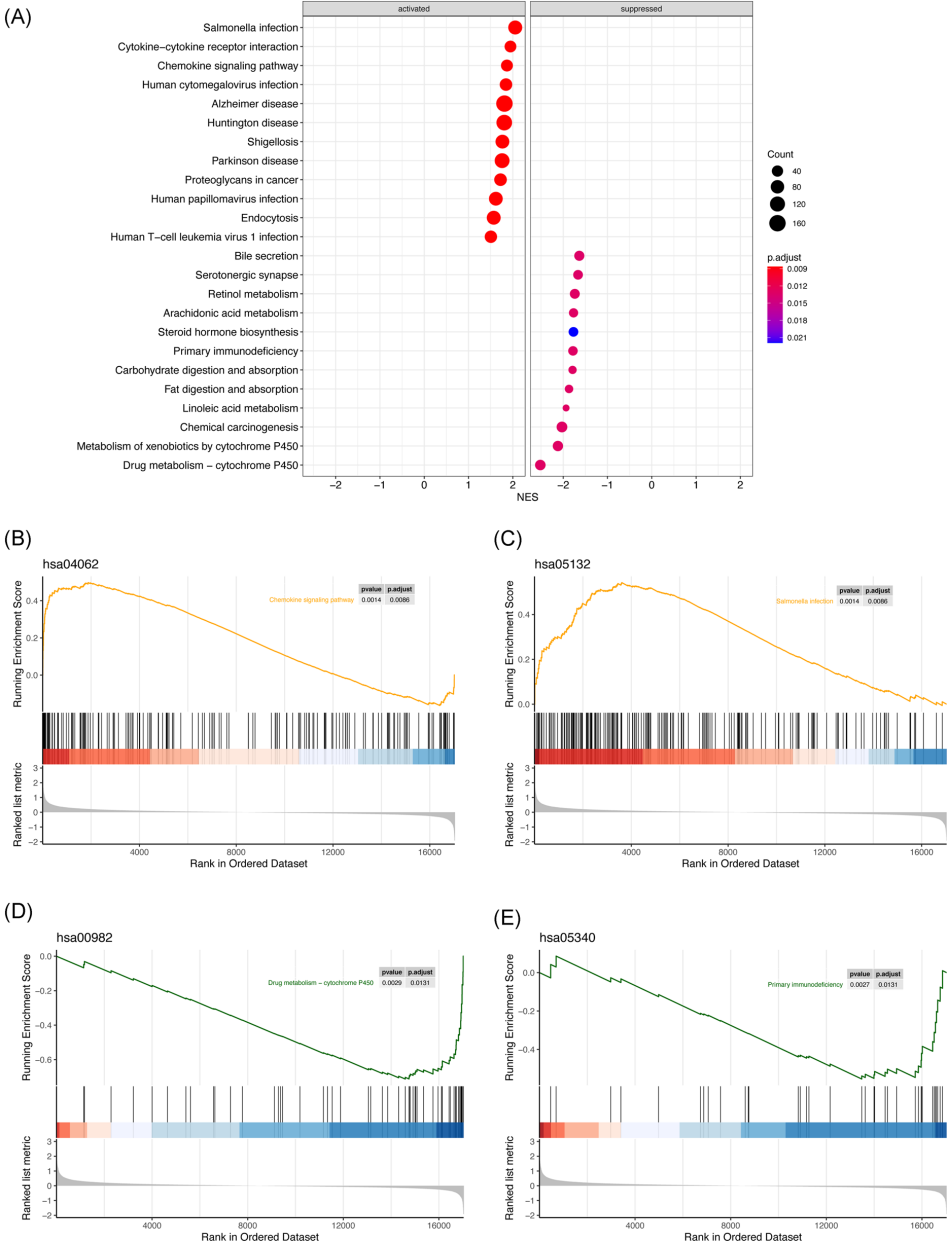
GSEA‐based KEGG enrichment analysis. (A) Remarkably enriched activated and suppressed KEGG pathways. The vertical items are the names of KEGG terms, and the *X*‐axis represents the normalized enrichment score (NES). The adjusted *p* value is shown as the depth of color. Circle size means gene counts in the graph. (B) The plots of GSEA‐based KEGG enrichment analysis of representative gene sets from activated pathway: Chemokine signaling pathway. (C) The plots of GSEA‐based KEGG enrichment analysis of  representative gene sets from activated pathway: Salmonella infection. (D) The plots of GSEA‐based KEGG enrichment analysis of representative gene sets from suppressed pathway: drug metabolism−cytochrome P450. (E) The plots of GSEA‐based KEGG enrichment analysis of representative gene sets from suppressed pathway: primary immunodeficiency. GSEA, gene set enrichment analysis; KEGG, Kyoto Encyclopedia of Genes and Genomes

As shown in Figure 2A, the 24 most prominent KEGG pathways, containing activated and suppressed pathways, were screened out. The absolute value of their normalized enrichment score was concentrated between 1 and 3. Among the activated pathways, “chemokine signaling pathway,” “Salmonella infection,” “human papillomavirus infection,” and “human T‐cell leukemia virus 1 infection” were connected to cellular immunity. However, suppressed pathways, such as “chemical carcinogenesis,” “metabolism of xenobiotics by cytochrome P450,” “drug metabolism—cytochrome P450,” and “serotonergic synapse,” were concentrated on drug metabolic process. The plots of GSEA‐based KEGG enrichment analysis of representative gene sets from activated pathways, including “chemokine signaling pathway” (adjusted *p* = .0086) and “Salmonella infection” (adjusted *p* = .0086), are shown in Figure 2B,C. Most of the upregulated genes were concentrated at the front of the sequence, which indicates that their upregulation was concentrated on the CD group. The GSEA‐based KEGG enrichment plots of representative gene sets from suppressed pathways, including “drug metabolism−cytochrome P450” (adjusted *p* = .0131) and “primary immunodeficiency” (adjusted *p* = .0131), are shown in Figure 2D,E, respectively. The majority of the upregulated genes were centered on the control group; therefore, the expression of this group of genes was inhibited in the disease group.

### 3.3 GO enrichment analysis of the significant DEGs

**Figure 3:**
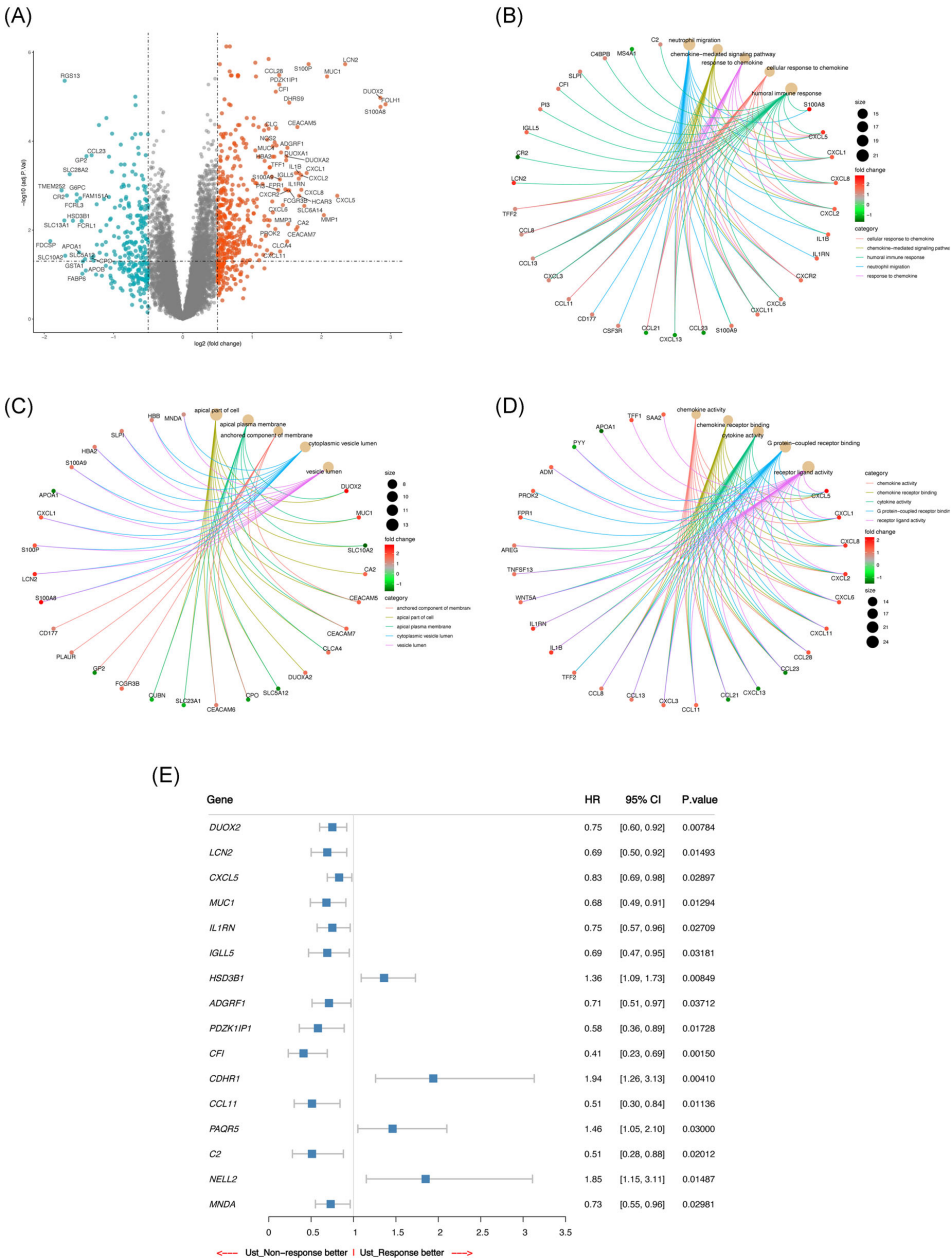
GO and univariate logistic analyses of significant DEGs in UST response. (A) Volcano plot of DEGs. DEGs in CD samples comparable to those in normal samples. Downregulated, upregulated, and nonsignificant genes are highlighted blue, red, and gray plots, respectively. The horizontal axis denotes the log2 (FC), and the vertical axis denotes—log10 (adjusted *p* value); The dots above the horizontal line represent the significant DEGs. (B) Top 5 GO terms in BP. Adjusted *p* < .05 was considered significant. (C) Top 5 GO terms in CC. Adjusted *p* < .05 was considered significant. (D) Top 5 GO terms in MF. Adjusted *p* < .05 was considered significant. (E) Random forest plot of genes that may be related to UST response. BP, biological process; CC, cellular component; CD, Crohn's disease; DEGs, differentially expressed genes; GO, Gene Ontology; MF, molecular function; UST, ustekinumab

The volcano plots of downregulated, upregulated, and nonsignificant genes in CD samples versus those in normal samples are shown in Figure 3A. The red plot represents the upregulated genes, and a plot far from the baseline indicates a more outstanding upregulation. The outstanding upregulated genes include S100A8, FOLH1, DUOX2, and LCN2. The blue plot represents downregulated genes, and the outstanding downregulated genes include FDCSP, SLC10A2, SLC13A1, and TMEM252. In BP, the top five most enriched GO terms are “neutrophil migration,” “chemokine‐mediated signaling pathway,” “response to chemokine,” “cellular response to chemokine,” and “humoral immune response.” Figure 3B shows that many genes, such as CXCL1, CXCL6, and CXCL8, play the role of a bridge. Some unique genes are also displayed. For example, IL1RN, CD177, and CR2 are related to only one GO term. Most of the genes were related to “chemokine response” and “humoral immune response.” The top five GO terms in CC include “apical part of cell,” “apical plasma membrane,” “anchored component of membrane,” “cytoplasmic vesicle lumen,” and “vesicle lumen” as shown in Figure 3C. The bridge genes include CEACM5, CEACM7, and CPO. The unique genes include CA2, DUOXA2, GP2, FCGR3B, FLAUR, and CD177. Most of the genes were connected with “apical plasma membrane.” Figure 3D shows the top five GO terms in MF, namely “chemokine activity,” “chemokine receptor binding,” “cytokine activity,” “G protein‐coupled receptor binding,” and “receptor–ligand activity.” The bridge genes include CXCL1, CXCL2, CXCL5. The unique genes comprise TFF1, SAA2, APOA1, PROK2, and FPR1. Most genes in MF were related to “receptor–ligand activity.”

### 3.4 Univariate logistic regression analysis

After conducting univariate regression analysis on the 122 significant DEGs, we obtained 16 potential predictors and visualized the results using a random forest plot. Figure 3E shows that HSD3B1 (HR 1.36, *p* = .00849), CDHR1 (HR 1.94, *p* = .00410), PAQR5 (HR 1.46, *p* = .03000), and NELL2 (HR 1.85, *p* = .01487) may be better predictors of UST response. However, DUOX2 (HR 0.75, *p* = .00784), LCN2 (HR 0.69, *p* = .01493), CXCL5 (HR 0.83, *p* = .0.2897), MUC1 (HR 0.68, *p* = .01294), IL1RN (HR 0.75, *p* = .02709), IGLL5 (HR 0.69, *p* = .03181), ADGRF1 (HR 0.71, *p* = .03712), PDZK1IP1 (HR 0.58, *p* = .01728), CFI (HR 0.41, *p* = .00150), CCL11 (HR 0.51, *p* = .01136), C2 (HR 0.51, *p* = .02012), and MNDA (HR 0.73, *p* = .02981) may be better predictors of UST nonresponse.

### 3.5 Multivariate predicative model

**Figure 4:**
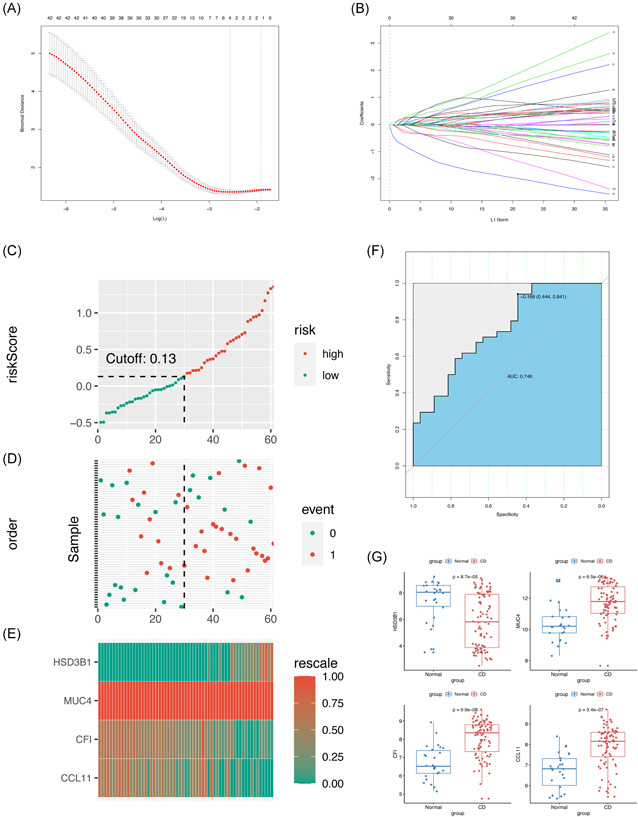
Training for the multivariate predictive model by LASSO regression and evaluation. (A) The tuning parameter (λ) selection in the LASSO model through tenfold cross‐validation was plotted as a function of log (λ). The *y*‐axis is for partial likelihood deviance, and the lower *x*‐axis for log (λ). The average number of predictors is represented along the upper *x*‐axis. Red dots indicate average deviance values for each model with a given λ, where the model is the best‐fit to data. (B) LASSO coefficient profiles of the 122 DEGs. The gray dotted vertical line is the value selected using tenfold cross‐validation in (A). (C) Distribution of risk score under the training set. (D) UST response of patients under the training set. The black dotted line represents the optimum cutoff point that divides patients into low‐ and high‐risk groups. (E) Heat map of the gene expression values of the final predictors under the training set. (F) ROC curves for patients under the training set. (G) Boxplot of the expression value of each gene in the predictive model. AUC, area under the curve; DEGs, differentially expressed genes; LASSO, least absolute shrinkage and selection operator; UST, ustekinumab

Figure 4A,B shows the results of the LASSO regression analysis of the 122 candidate DEGs. A multivariate logistic regression equation, which was composed of four genes and has the predictive ability for UST response, was built. The final predictive model using LASSO regression was composed of *HSD3B1* (regression coefficient = 0.10506761, *p* = .000087), *MUC4* (regression coefficient = −0.01419220, *p* = .0000065), *CF1* (regression coefficient = −0.41004617, *p* = .000000099), and *CCL11* (regression coefficient = −0.01087779, *p* = .00000034) as shown in Figure 4G. Subsequently, an individual risk score was calculated for each patient in the training set through the multivariate predictive model. We categorized the patients into high‐score or low‐score groups according to the optimal cut‐off point determined by the highest sensitivity and specificity of the ROC curve (Figure 4C). Patients with scores ≥ 0.13 were assigned to the high‐score group, while the remaining patients belonged to the low‐score group. Figure 4D shows the actual UST response of patients in the training set. Patients who scored high are more likely to have a better response to UST, whereas patients with low scores are more likely to poorly respond to UST. Figure 4E describes the expression level of the four genes of the prediction equation in each sample. *HSD3B1* and *MUC4* were expressed evenly in every sample in the training set. Additionally, *CF1* and *CCL11* expressed some differences in different samples; however, the overall expression is still consistent in the training set. Figure 4F shows the ROC curve for patients under the training set. In this figure, the area under the ROC curve (AUC) of the predictive model for UST response is 0.746, which indicates that the predictive ability of the model is good. Figure 4G shows the Boxplot of the expression value of each gene in the predictive model. The figure shows that HSD3B1 (*p* = .000087) was upregulated in the normal group and downregulated in the patient group. *MUC4* (*p* = .000006.5), *CF1* (*p* = .000000099), and *CCL11* (*p* = .00000034) were upregulated in the patient group but downregulated in the normal group.

### 3.6 Evaluation for the multivariate predictive model

**Figure 5:**
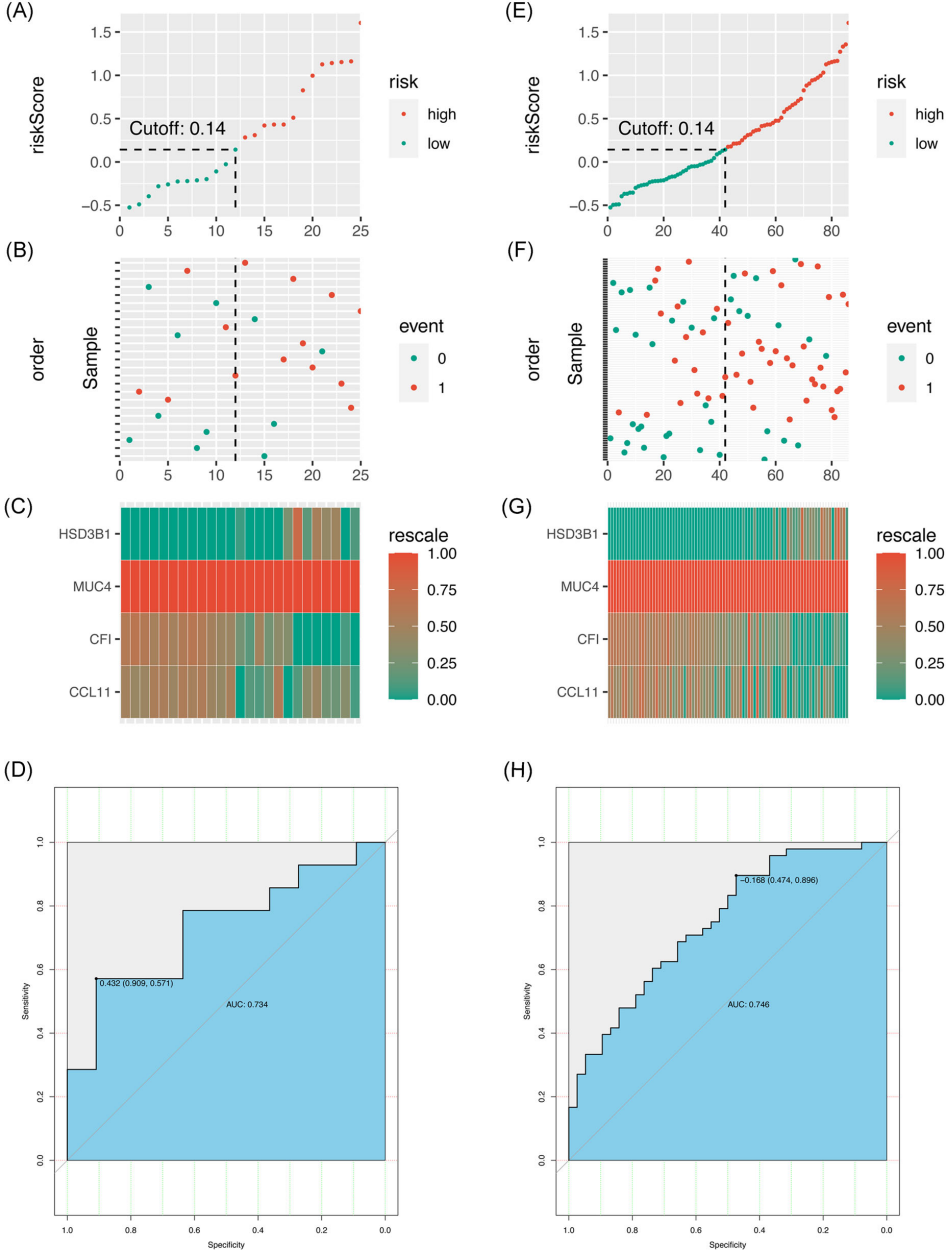
Testing the multivariate predictive model. (A–D). Testing the model under the testing set. (A) Distribution of risk score under the testing set. (B) UST response of patients under the testing set. (C) Heat map of the gene expression values of the final predictors under the testing set. (D) ROC curves for patients under the testing set. (E–H). Testing the model under the total dataset. (E) Distribution of risk score under the total set. (F) UST response of patients under the total set. (G) Heat map of the gene expression values of the final predictors under the total set. (H) ROC curves for patients under the total set. ROC, receiver operator characteristic; UST, ustekinumab

We performed the same analyses in the testing set and the total dataset to verify the results in the training set. The risk score of each patient in the testing set and total dataset was calculated using the multivariate predictive model. The cut‐off score was 0.14, which is close to the value of the training set. The results are shown in Figure 5A,E. The UST responses of patients under the testing set and total dataset are shown in Figure 5B,F, respectively. The expression profiles of HSD3B1, MUC4, CF1, and CCL11 in the two datasets (Figure 5C,G) are similar to those in the training dataset. The AUCs in the testing set and total dataset were 0.734 and 0.746, respectively. This observation confirmed the predictive power of the final model in the testing set (Figure 5D,H). Therefore, the predictive model has a good prediction for the UST response of patients with CD.

## 4 DISCUSSION

We searched all datasets related to inflammatory bowel disease (IBD) in GEO, and find only this dataset (GSE112366) includes UST using. To reduce data bias, all samples were divided randomly to training (70%) and testing (30%) sets using the “createDataPartition” function in the R package “caret.” This function can keep each categorical variable of the data in the subset consistent with the original proportion of the overall data. In the present study, we performed the bioinformatics method to acquire the significant genes related to UST response in patients with CD. Furthermore, we constructed an independent and efficient predictive model. Some related genes and predictive models of IBD have been reported in previous studies using bioinformatics analysis.^25^, ^28^, ^29^, ^30^, ^31^ However, these studies focused on IBD and did not further discuss CD or UC separately. Besides, Leal et al.^32^ have elucidated inflammatory mediators in patients with CD who are unresponsive to anti‐TNFα therapy. However, no information on the bioinformatics analysis of the UST response of patients with CD was available. This study is the first to explore the genes with predictive power for UST response using bioinformatic analysis and the first to construct a predictive model for patients with CD who intend to try UST treatment. This study found by GSEA‐based KEGG analysis that most of the activated pathways are in connection with cellular immunity, which is in agreement with previous reports.^28^, ^31^, ^33^, ^34^ Besides, we uncovered the potential functions of DEGs using GO analysis. The most significantly enriched GO terms among BP and MF pathways are related to inflammation. This finding is also consistent with previous studies; therefore, the results of the GO analysis in our study were reasonable.^32^, ^35^, ^36^, ^37^, ^38^

We first constructed a predictive model through applying LASSO regression analysis for candidate DEGs. The model, which was composed of *HSD3B1, MUC4, CF1*, and *CCL11*, showed good predictive capacity for drug response. Compared with multivariate COX regression, which is chosen to build a multivariate model by focusing on several variables, LASSO regression is preferably suitable for the regression of massive and multivariate variables.^22^, ^39^, ^40^, ^41^, ^42^ Herein, we adopted LASSO regression to obtain the final important predictors to build the predictive model. Subsequently, this study showed that the AUC manifested favorable sensitivity and specificity in the training set. Moreover, the AUCs of the multivariate predictive model in the test group and the total dataset were similar, which indicates that the predictive model has a favorable performance and could provide a potential therapeutic strategy for decision making on the use of UST treatment among patients with CD.

As one of the four most powerful predictors, *MUC4* is transmembrane mucin universally expressed in the small and large intestines and plays a critical role in cell proliferation and the differentiation of epithelial cells by inducing the specific phosphorylation of ERBB2. *MUC4* is commonly disturbed in the intestinal samples of patients with IBD; thus, it acts as a crucial player in IBD.^8^, ^43^, ^44^, ^45^, ^46^, ^47^, ^48^ Das^49^ demonstrated that *MUC4* drives intestinal inflammation and inflammation‐associated tumorigenesis using a novel Muc4−/− mouse model. However, the occurrence of IBD is likely related to the disturbed epithelial cells of the intestines.^27^, ^50^ As another predictor in the model, *CCL11* is a potent eosinophil chemoattractant that is constitutively expressed in the small intestine and colon. Besides, CCL11 is highly expressed in active CD, contributes to tissue eosinophilia, and regulates intestinal inflammation.^51^, ^52^ *HSD3B1*, as a steroidogenesis gene, is associated with GC resistance.^53^ *CF1* is associated with metabolism.^54^ Interestingly, the participation of *HSD3B1* and *CF1* in CD was unknown and first unveiled to be related to the UST responsiveness of patients within our study.

This study has several limitations. First, the degree of UST response of each patient was not reported in detail. Besides, as a clinical predictive model, the model has not yet been validated by external data. The model will be validated in our future study.

## 5 CONCLUSIONS

Our study provided new insight into the expression of genes related to the UST response of patients with CD. This study unveiled the important DEGs in this field and built a powerful predictive model, which could possibly provide valuable data sources for further basic and clinical studies in the future.

## CONFLICT OF INTERESTS

The authors declare that there are no conflict of interests.

## AUTHOR CONTRIBUTIONS

Yufang Wang designed the study. Manrong He, Chao Li, Yingxi Kang, and Yongdi Zuo prepared the data. Wanxin Tang and Chao Li analyzed the data. Wanxin Tang, Manrong He, and Yufang Wang wrote the manuscript. All authors read and approved the final manuscript.

## Data Availability

The datasets in the current study come from the GEO database: GSE112366.
